# Supplementary material for: Plasticity in gustatory and nociceptive neurons controls decision making in C. elegans salt navigation
Source: Commun Biol. 2021 Sep 9;4:1053. doi: 10.1038/s42003-021-02561-9 (PMC8429449; doi:10.1038/s42003-021-02561-9)
Supplement: Supplementary file 2 — Supplementary Information [file 42003_2021_2561_MOESM2_ESM.pdf]

Plasticity in gustatory and nociceptive neurons controls decision making in *C. elegans* salt navigation

Martijn P.J. Dekkers, Felix Salfelder, Tom Sanders, Oluwatoroti Umuerri, Netta Cohen, Gert Jansen

Supplementary information

Supplementary Figures 1-8
Supplementary Table 1
Supplementary Methods

Supplementary figures

Supplementary Fig. 1: Pre-exposure to NaCl significantly reduces chemotaxis to NaCl and is time dependent.

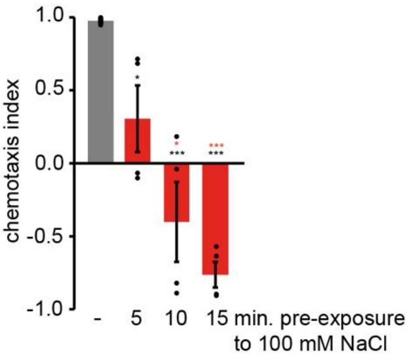

Wild type animals were tested for chemotaxis to 25 mM NaCl after exposure to 100 mM NaCl for 5-15 min. (red bars), or as a control to a buffer containing no NaCl, for 15 min. (gray bar; 5 or 10 min. wash in a buffer without NaCl gave similar results as 15 min. wash). 5-15 min. pre-exposure resulted in a significant decrease of chemotaxis to NaCl (black * $p<0.05$, *** $p<0.001$, compared to no NaCl). 10 or 15 min. pre-exposure resulted in a significantly stronger reduction of chemotaxis (red * $p<0.05$, *** $p<0.001$, compared to 5 min. pre-exposure). 15 min. pre-exposure did not significantly reduce chemotaxis further, compared to 10 min. pre-exposure ($p>0.05$). Indicated are the average chemotaxis index (±SEM) of four independent assays. Individual datapoints have been indicated as dots.

Supplementary Fig. 2: Pre-exposure to NaCl does not affect chemotaxis to higher NaCl concentrations.

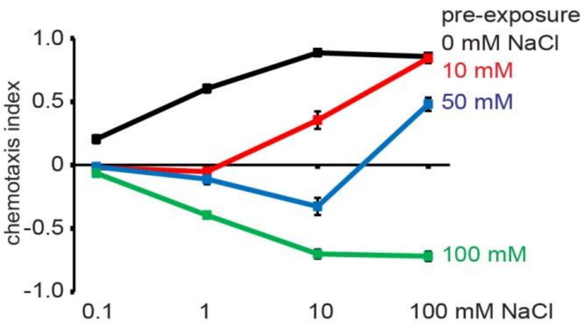

Wild type animals were tested for chemotaxis to 0.1, 1, 10 or 100 mM NaCl after exposure to 0, 10, 50 or 100 mM NaCl for 15 min. Pre-exposure strongly affected chemotaxis to NaCl concentrations lower or similar to the pre-exposure concentration but did not affect chemotaxis to higher concentrations: chemotaxis to 10 mM was significantly different from that to 100 mM for animals pre-exposed to 10 mM NaCl ($p<0.001$) or to 50 mM ($p<0.001$), but not after pre-exposure to 0 or 100 mM NaCl ($p>0.05$). Indicated are the averages of 18 assays ± SEM.

Supplementary Fig. 3: Desensitization of ASEL in various mutant strains.

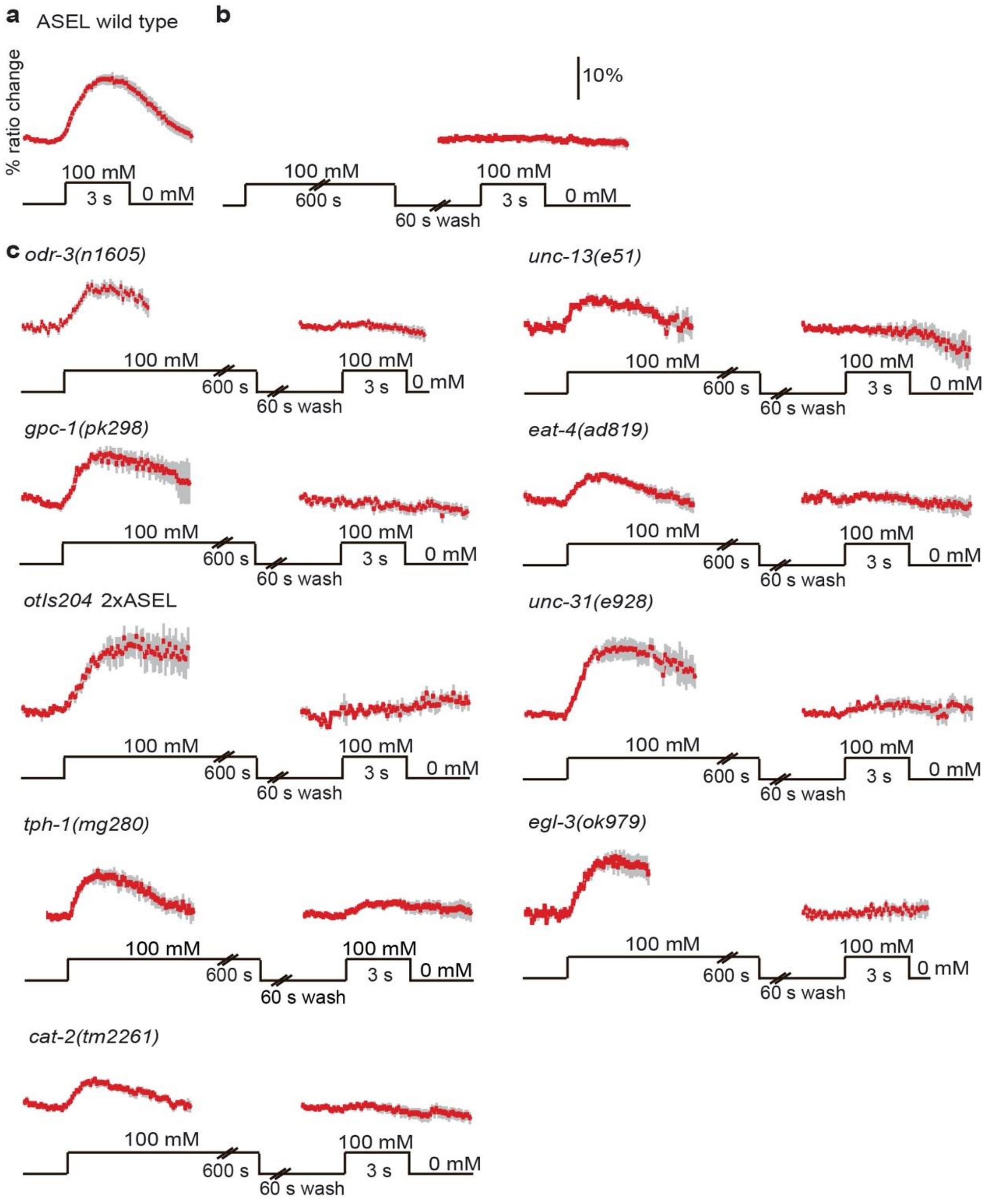

a Average Ca^{2+} transients (± SEM) of naïve wild type animals in response to a 3 seconds exposure to 100 mM NaCl (data from Fig. 1). **b** Average Ca^{2+} transients (± SEM) of wild type animals in response to 100 mM NaCl after 10 minutes exposure to 100 mM NaCl (data from Fig. 3). **c** Average Ca^{2+} transients (± SEM) in ASEL of *odr-3(n1605)* (n=7 animals), *gpc-1(pk298)* (n=7), *otIs204* (n=4), *tph-1(mg208)* (n=9), *cat-2(tm2261)* (n=9), *unc-13(e51)* (n=7), *eat-4(ad819)* (n=8), *unc-31(e928)* (n=8) and *egl-3(ok979)* (n=7) animals in response to 100 mM NaCl, either naïvely or after 10 minutes exposure to 100 mM NaCl. The average maximum ratio changes of none of the mutants was significantly different from that of wild type animals (p>0.05).

Supplementary Fig. 4: Sensitization of ASER does not involve neuropeptides, dopamine, or serotonin.

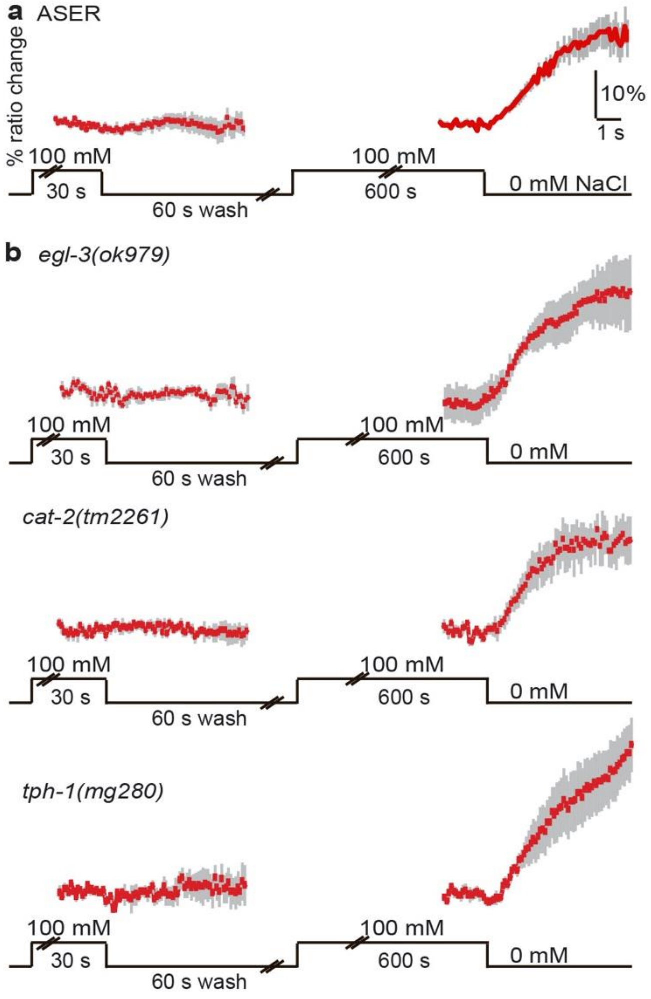

a Average Ca^{2+} transients (± SEM) in ASER in wild type animals in response to a decrease in NaCl concentration from 100 mM to 0 mM after 30 or 600 seconds exposure (data from Fig. 5). **b** Average Ca^{2+} transients (± SEM) in ASER neurons of *egl-3(ok979)*, *cat-2(tm2261)* and *tph-1(mg280)* animals in response to a decrease in NaCl concentration from 100 mM to 0 mM after 30 or 600 seconds exposure. 30 seconds exposure to 100 mM NaCl did not result in a response in ASER, but 10 minutes exposure did result in a Ca^{2+} response. *egl-3(ok979)*: n=4 animals, *cat-2(tm2261)* n=4, *tph-1(mg280)* n=5. The average maximum ratio changes of none of the mutants was significantly different from that of wild type animals ($p>0.05$).

Supplementary Fig. 5: ASEL Ca^{2+} response is faster than ASER response.

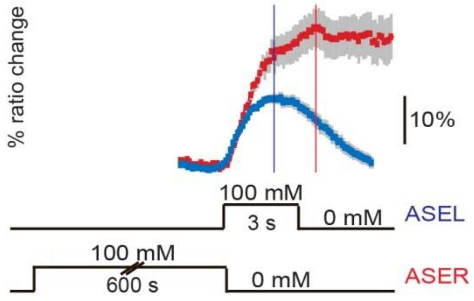

Superimposed average Ca^{2+} transients of fully sensitized ASEL (blue) and ASER (red) neurons of animals exposed to 100 mM increases or decreases (600s pre-exposed), taken from Fig. 1 and 2 respectively. Vertical lines denote time of peak depolarization.

Supplementary Fig. 6: Model worm trajectories on quadrant NaCl-choice assay.

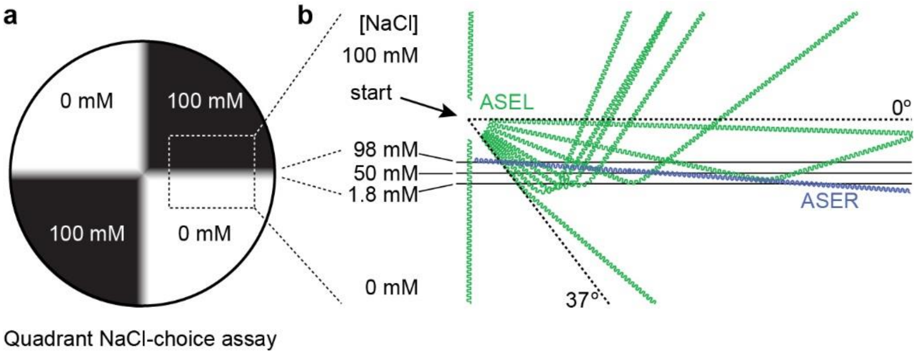

a Schematic of quadrant NaCl-choice assay[9]. Animals are delivered in the middle of a plate divided in 4 quadrants filled with buffered agar, 2 quadrants with e.g. 100 mM NaCl, 2 quadrants without NaCl, separated by plastic spacers. Just before the assay the area between the quadrants is filled with agar, resulting in a very steep gradient from 100 to 0 mM NaCl (see Supplementary methods). **b** Sample trajectories of virtual worms with either virtual ablations of ASER (ASEL only, in green) or ASEL (ASER only, in blue), initiated (start) in the vicinity of a steep salt transition between 100 and 0 mM NaCl (modeled after the quadrant assay). NaCl concentrations in the transition area have been indicated. For sufficiently shallow angles of attack (down the salt gradient), ASEL successfully mediates strong steering up the salt gradient. The effect of ASER on the steering is negligible. Simulations with virtual ASEL ablations yielded a maximum change of orientation below 2° and only in trajectories close to perpendicular to the salt gradient.

Supplementary Fig. 7: Robustness of the model performance in the virtual quadrant assay is not reduced by ASE (de)sensitization.

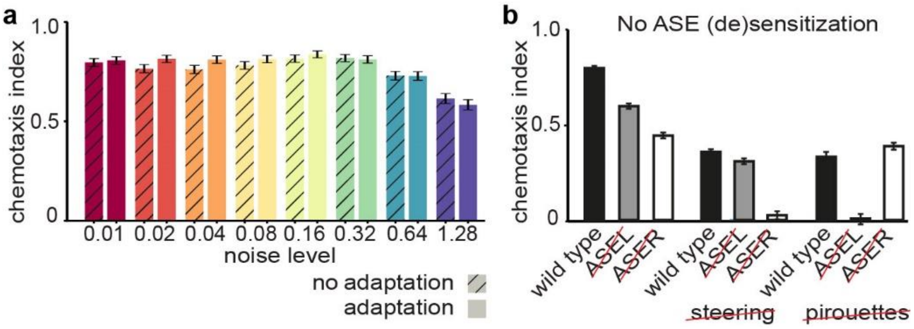

a Populations of animals display similar CI distributions with and without ASE (de)sensitization. The CI (mean and standard deviation) for 'noisy' populations of $n = 500$ animals. In each animal within each population, ASE parameters were perturbed with the specified noise amplitude (see Supplementary Methods). Performance was measured by the CI of the population after 10 minutes in the quadrant assay. CI levels remained steady with noise amplitudes up to σ =0.32. Regardless of CI performance, model worms with (de)sensitization (test group) performed at least as well as those without sensory adaptation (control group). For σ >0.32 the performance dropped in both test and control groups. **b** ASE (de)sensitization does not contribute to the asymmetric functions of the ASE neurons in our computational model. Chemotaxis index in the virtual quadrant assay of animals with fully sensitized ASEL and ASER. Ablating ASEL or ASER and disabling steering or pirouettes have very similar effects as in animals with wild type ASE (de)sensitization (Fig. 7g).

Supplementary Fig. 8: Exploration behavior over time in the virtual spot assay.

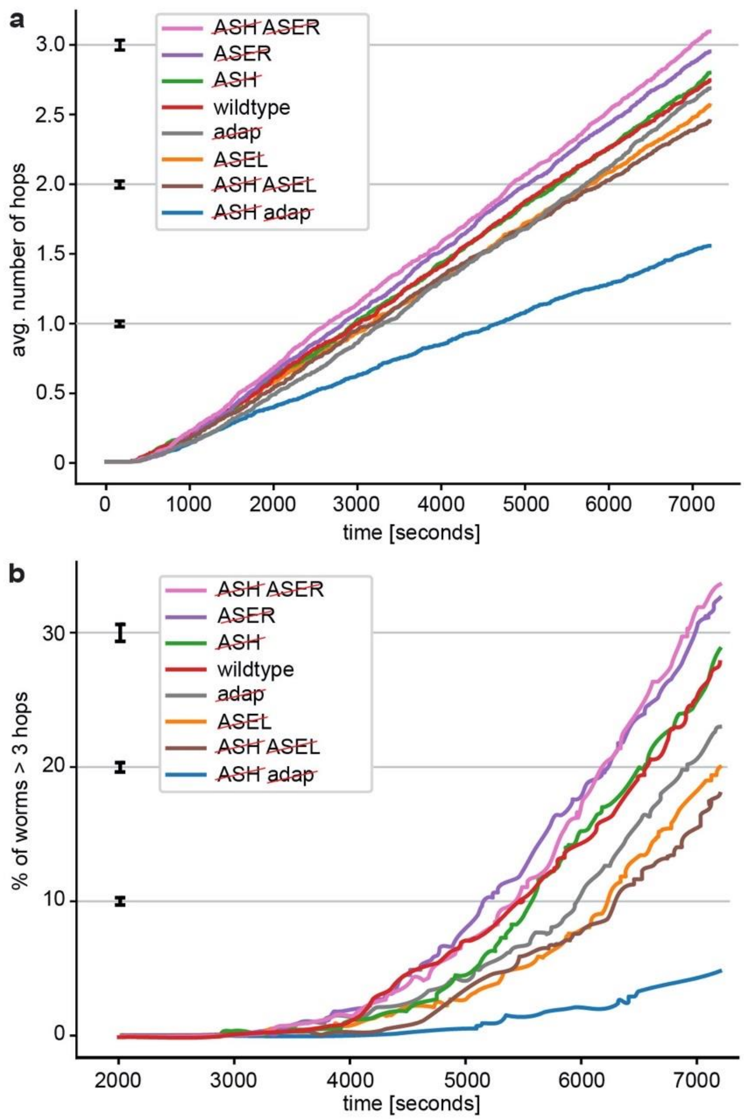

The time evolution of the hop counts from Fig. 9. A hop from one spot to another is registered once a worm trajectory meets a spot for the first time, after leaving another spot. **a** The average number of hops per worm over time, of wild type worms and worms in which specific cells were ablated (e.g. ~~ASER~~) or in which ASEL and ASER are always fully sensitized (~~adap~~). The average number of hops is hardly affected by ablating ASH or ASEL at all times. **b** Fraction of worms that hopped at least three times.

Supplementary Table 1. ASEL, ASER and ASH Ca^{2+} responses

Neuron	Figure	Pre-exp. time (s)	Wash time (s)	Exposure time (s)	Exposure [NaCl] (mM)	n	Response Responding	Response Index[1]	Rise time (s) Mean ± SD	Rise time (s) Range
ASEL	1a,b			3	10	17	10	0.59	1.6 ± 0.6	0.5 - 2.8
				3	100	42	37	0.88	1.9 ± 0.7	0.7 - 3.8
				3	200	16	9	0.56	1.9 ± 0.7	1.0 - 3.6
				3	300	17	15	0.88	2.0 ± 0.7	1.0 - 3.4
				3	500	11	8	0.73	1.5 ± 0.6	0.5 - 2.3
ASEL	3a,b	60	60	3	100	13	10	0.77	2.1 ± 0.9	1.4 - 4.2
		120	60	3	100	9	8	0.89	2.3 ± 0.9	1.0 - 3.8
		300	60	3	100	8	4	0.50	1.4 ± 1.2	0.4 - 3.3
		600	60	3	100	16	0	0.00		
ASEL	3e,f	600	120	3	100	8	6	0.75	3.0 ± 1.2	1.5 - 4.8
		600	300	3	100	6	5	0.83	2.9 ± 1.3	1.1 - 4.9
ASH	1d,e			3	100	18	5	0.28	2.7 ± 0.7	1.8 - 3.4
				3	200	18	5	0.28	1.7 ± 0.7	0.7 - 2.6
				3	300	22	16	0.73	2.4 ± 0.6	1.7 - 4.2
				3	500	19	16	0.84	2.3 ± 0.6	1.5 - 3.6
ASH	4a,b	600		3	200	17	16	0.94	2.6 ± 0.4	1.8 - 3.5
		600		3	300	12	10	0.83	3.0 ± 0.3	2.6 - 3.6
		600		3	500	24	19	0.79	2.9 ± 0.7	2.2 - 4.6
ASER	1c			3	10	8	0	0		
				3	100	30	0	0		
				3	200	5	0	0		
				3	300	9	0	0		
				3	500	4	0	0		
ASER	2a,b			30	100	8	1	0.13	8.3	8.3
				60	100	15	10	0.66	6.0 ± 4.3	0.4 - 13.0
				120	100	4	4	1.00	5.5 ± 3.0	2.0 - 8.5
				300	100	5	3	0.60	3.4 ± 0.9	2.2 - 4.4
				600	100	29	25	0.86	6.2 ± 3.4	1.8 - 13.5

[1]. The fraction of animals responding has been indicated as the response index (# animals responding / # animals tested (n)).

Supplementary methods

Computational model

A computational simulation framework was developed to study *in silico* the behavior of single model worms confronted with the quadrant assay. A variation of this computational model has previously been used to model a *C. elegans* decision making assay[1]. Our model of the animal assumes that during locomotion, the body follows the head, allowing us to focus on sensory-motor control of a point worm. Thus, at each point in time, animals are modeled as a point $(x(t), y(t))$ representing the head heading towards an angle, $\theta(t)$. The head moves at a fixed speed, $v = 0.22$ mm/s, resulting in an overall body speed of $v = 0.1$ mm/s. Our model *C. elegans* sense their environment using a simplified nervous system which dynamically controls the direction of locomotion[1,2].

To study gustatory plasticity and the effects of salt adaptation on navigation, our model circuit contained three sensory neurons (ASEL, ASER and ASH) that synapse directly onto a simplified motor system (Supplementary Fig. 9). The motor output consists of undulations that are modulated by the sensory system to generate steering, in addition to instant turning events representing pirouettes. All neuronal equations and parameters, including the sensory responses and motor neuron dynamics were constrained by Ca^{2+} imaging data[3,4] and published behavioral data[4-8].

Supplementary Fig. 9: Schematic of the computational model.

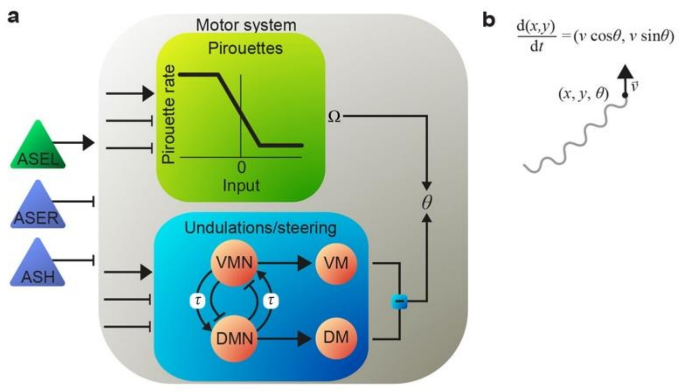

a The model circuit contains three sensory neurons, ASEL, ASER and ASH (triangles), and a motor circuit (grey box). Each sensory neuron projects onto all three components of the motor circuit (Pirouettes, VMN, DMN). A motor command unit (green box) regulates the pirouette rate as a function of sensory input. Positive synaptic input suppresses the probability of a pirouette; negative input enhances it. A pirouette (Ω) is modeled as an instantaneous, random reorientation. A half-center oscillator circuit (blue box) generates undulations and steers the worm in response to synaptic inputs from sensory neurons: Two motor neurons (VMN, DMN) drive model muscles (VM, DM) on the respective sides of the body. Each delay unit τ and muscle (VM, DM) in the motor circuit is modeled as an implicit neuronal unit. The output of the sensory motor circuit is an instantaneous orientation, $\theta(t)$. **b** Visualization of the worm coordinates in time. An animal at time t is represented by a point, $(x(t), y(t))$, in a two-dimensional space that moves with fixed speed, v, in a direction, $\theta(t)$.

We followed an incremental approach for parameter tuning of the model. First, sensory neuron rate parameters $(\alpha_i, \beta_i, \gamma_i)$ were manually fit to match Ca^{2+} imaging traces of naïve animals (Supplementary Fig. 11). In Supplementary Figure 10, α_i determines the steepness of the fast conductance; γ_i determines the steepness of the slow conductance; and α_i/β_i determines the saturation amplitude of F (in arbitrary units). Together, these parameters determine the current profile $(\mathrm{F} - \mathrm{S})$. The current-voltage relation (Equation E1 below) was not fit (parameter values used were the

same as in as Ghosh *et al.* (2016)[1]). The motor circuit (synaptic weight parameter set in Supplementary Table 2) was independently modeled to obtain realistic undulations and pirouette rates. Modeling salt navigation behaviors only required setting the synaptic weights from salt sensory neurons onto the motor circuit (to match observed steering and turning rates in the quadrant assay). This modeling approach ensures that the model is maximally constrained and should enhance its predictive potential. Once the model was fully parametrized, it was tested, first on the quadrant assay, and second, on a new *in silico* spot assay. Only the base pirouette rate was adjusted in the spot assay, in agreement with observations on related assays[8].

Model neurons

We modelled all neurons as leaky integrators (with arbitrary units)

$$\tau_\mathrm{m}\frac{\mathrm{d}V_i}{\mathrm{d}t} = -V_i + V_{0,i} + \sigma(I), \qquad \text{(E1)}$$

where V_i is a voltage like variable for neuron i, also referred to here as neuronal activation; τ_m is a neuronal time constant; finally, $V_{0,i}$ represents the resting potential (set throughout to zero). The function $\sigma(I)$ is a sigmoid defined as

$$\sigma(I) = \tanh(bI), \qquad \text{(E2)}$$

where b is a gain parameter and the input current is denoted $I = I(t)$ (with the implicit neuronal index i, Supplementary Table 2). Thus, a neuron's activation ranges from -1 to 1 and converges to $\sigma(I)$ with a timescale of τ_m for fixed input, I. This formulation is borrowed from rate neuron models and exhibits thresholding and saturation of activation, as observed in *C. elegans* sensory neurons[3,4,9]. As the bilateral ASHL/ASHR neuronal pair is coupled by gap junctions[10] and ASH Left and Right neurons are known to respond identically[4], the ASH neuron pair was collapsed into a single model neuron.

Supplementary Table 2. Neuronal parameters and synaptic weights.

Neuronal parameters	Value	Description
τ_m	0.5 s	Neuronal time constant
$V_{0,i}$	0	Resting potential
b	2	Neuronal gain
**Synaptic weights**	**Value**	**Description**
$W_{\mathrm{ASEL,M}}$	0.5	ASEL onto DMN and VMN
$W_{\mathrm{ASER,M}}$	-0.5	ASER onto DMN and VMN
$W_{\mathrm{ASH,M}}$	0.5	ASH onto DMN and VMN
$W_{\mathrm{ASEL,\Omega}}$	-0.25	ASEL onto Ω
$W_{\mathrm{ASER,\Omega}}$	-0.25	ASER onto Ω
$W_{\mathrm{ASH,\Omega}}$	1	ASH onto Ω
$W^{+}_{\mathrm{D,V}}$	0.88	DMN to VMN excitation (to, from hidden neuron)
$W^{+}_{\mathrm{V,D}}$	0.88	VMN to DMN excitation (to, from hidden neuron)
$W^{-}_{\mathrm{D,V}}$	-1.4	DMN to VMN inhibition
$W^{-}_{\mathrm{V,D}}$	-1.4	VMN to DMN inhibition

The input current *I* sums over all synaptic and sensory contributions, $I = I_{\mathrm{syn}}(t) + I_{\mathrm{sens}}(t)$ (E3), where, assuming graded synaptic transmission, $I_{\mathrm{syn}} = \sum_j W_{ji} V_j$ (E4), is a weighted sum over all presynaptic neuron activations and I_{sens} denotes the stimulus response current in sensory neurons (given in Equations (E5)–(E7)). In line with the unrectified nature of graded synapses in *C. elegans*[11], hyperpolarizing a neuron will effectively reverse the polarity of its efferent synaptic transmission (with hyperpolarizing and depolarizing postsynaptic transmission across excitatory and inhibitory synapses, respectively).

Sensory neurons

Our model focuses on sensory responses to NaCl. In this study we looked exclusively at ASEL, ASER and the ASH sensory neuron pair. Ca^{2+} imaging data has shown that multiple sensory neurons in *C. elegans* respond to a change in a stimulus, rather than to the stimulus intensity directly. The response often approximates a time-filtered time derivative over the stimulus input[3,4,12,13]. Moreover, in many cases, changes in the stimulus level selectively produce a rectified depolarizing response with a characteristic rise time, followed by a slower decay to rest. In particular, ASEL and ASH neurons respond with a transient depolarization to increases in NaCl concentration (in the case of ASH, only to high concentrations above ~200 mM indicating a response to high osmolarity, rather than NaCl *per se*)[3]. In addition, ASER and ASH show a transient depolarization in response to decreases in the NaCl concentration[4].

The biphasic nature of the derivative-like responses seen in *C. elegans* sensory neurons suggests a model in which sensory neurons use two driving forces or conductances with opposite effect and a separation of timescales[13]. Importantly, the characteristic rise and decay times may be neuron specific (and possibly stimulus specific). Although detailed electrophysiological characterization is not yet available for these neurons, Ca^{2+} imaging with a variety of indicators[3,4,14], including our own data here, indicates that the ASER response (in particular the associated decay time) to a decrease in NaCl is much slower than the ASEL response to a NaCl increase.

In our model, the sensory contribution to the neuronal activity is denoted by a current, I_{sens}. We modeled sensory responses using a fast component, F, and a delayed rectifying component, S (Supplementary Fig. 10a). The sensory current I_{sens} is given by the difference between the fast and slow components, subject to either positive rectification (in ASEL), or reversal (ASER) depending on the cell. We represent this additional transformation generically as

$$I_{\mathrm{sens}} = f_i(F - S) \quad \text{(E5)}$$

The function, $f_{\mathrm{ASEL}}(x) = \max(0, x)$, enables ASEL to respond only to NaCl concentration increases; $f_{\mathrm{ASER}}(x) = -x$ allows ASER to respond to NaCl concentration decreases. The absence of rectification in ASER captures the effect of hyperpolarization in response to concentration increases. ASH is modeled without rectification or reversal, $f_{\mathrm{ASH}}(x) = x$. Fast and slow components evolve according to

$$\frac{\mathrm{d}F}{\mathrm{d}t} = D_i \alpha_i \max(0, C_{\log} - C_0) - \beta_i F, \quad \text{(E6)}$$

$$\frac{\mathrm{d}S}{\mathrm{d}t} = \gamma_i (F - S). \quad \text{(E7)}$$

Supplementary Fig. 10. Diagram of a sensory neuron and its response.

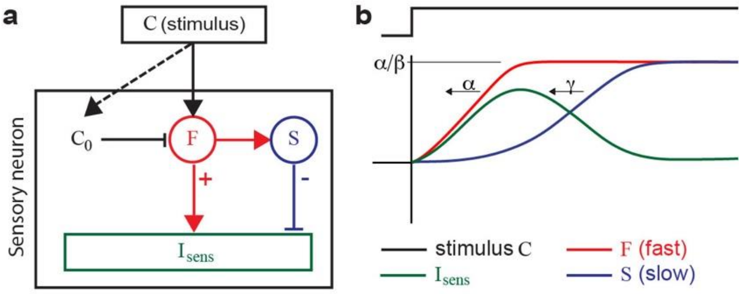

a A stimulus C evokes a rapid response, F, which in turn drives a slow, delayed response, S. The response F is subject to a threshold, C_0, depicted here as a bias input. The current I_{sens} is a function over the difference, $F - S$, such that S acts as a delayed rectifier. In ASEL, $C_0 \geq 0$ is a slowly adapting baseline that desensitizes the sensory neuron to the ambient stimulus strength (dashed arrow). **b** Schematic traces illustrating the response I_{sens} to a stimulus, C, given by the difference, $F - S$, (where $C_0 = 0$). α_i determines the steepness of the fast conductance; γ_i determines the steepness of the slow conductance; and α_i/β_i determines the saturation amplitude of F (in arbitrary units).

The fast component, F, responds to changes in the NaCl concentration with a characteristic rate β_i. The slow component, S, follows F with a delay, producing the transient response (Supplementary Fig. 10b). We used Ca^{2+} imaging traces under fully sensitized conditions to calibrate the global timescale τ_{m} (in compliance with the Motor circuit, Supplementary Fig. 9), as well as to fit the timing coefficients α, β and γ for the ASEL response (Supplementary Fig. 11a).

Supplementary Fig. 11. Model sensory neuron responses.

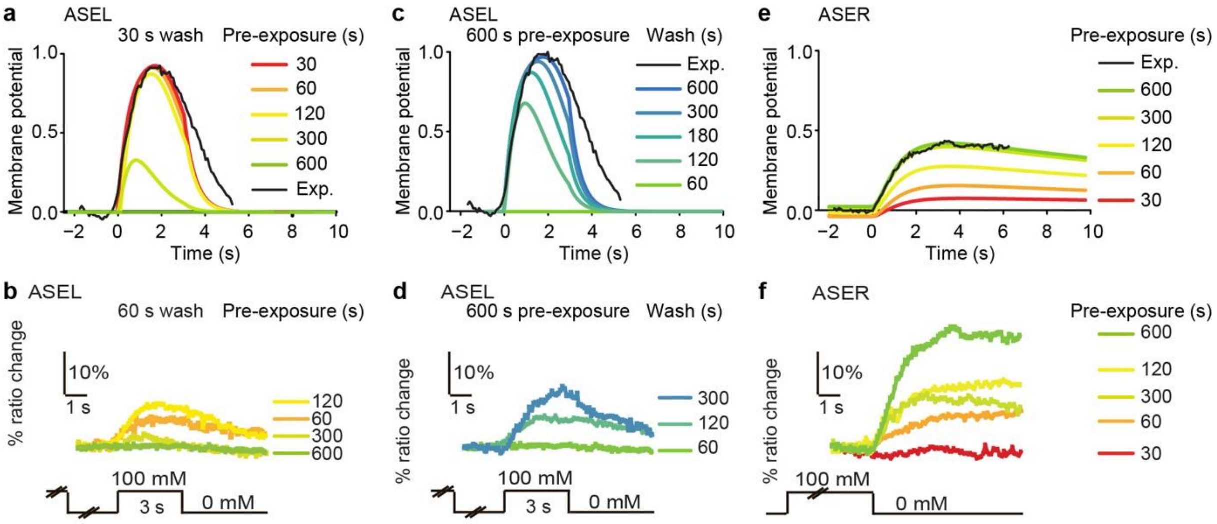

a Model ASEL responses to 3 seconds exposure to 100 mM NaCl (starting at 0 seconds) after pre-exposure of varying durations to 100 mM NaCl and a 30 seconds wash. A representative Ca^{2+} imaging trace (30 seconds pre-exposure to 100 mM NaCl, 30 seconds wash) in black, with vertical scaling to the amplitude of the corresponding model trace. **b** *In vivo* counterpart of (a): average Ca^{2+} transients in ASEL in response to 100 mM NaCl after 60-600 seconds pre-exposure and 60 seconds wash. Data from Fig. 3a. **c** ASEL responses to 3 seconds exposure to 100 mM NaCl after 600 seconds pre-exposure to 100 mM NaCl and a wash of varying durations. **d** *In vivo* counterpart of (c): average Ca^{2+} transients in ASEL in response to 100 mM NaCl after 600 seconds pre-exposure and 60-300 seconds wash. Data from Fig. 3a,e. **e** ASER responses to a decrease in NaCl from 100 to 0 mM (at 0 seconds) after pre-exposure to 100 mM NaCl of varying durations. A representative Ca^{2+} imaging trace (600

seconds pre-exposure to 100 mM NaCl) in black, with vertical scaling to the amplitude of the corresponding model trace. **f** *In vivo* counterpart of (e): average Ca^{2+} transients in ASER after 30-600 seconds pre-exposure to 100 mM NaCl. Data from Fig. 2a.

The order of magnitude for gain factor b was chosen so the response is clipped within the range of input current variations induced by pre-exposure (see below). We used b to tune the shape of the head trajectory (Supplementary Fig. 12a). For parsimony, we used the same gain factor and neuron timescale in all motor and sensory neurons. The timing coefficients for ASER where chosen similarly, to match the Ca^{2+} imaging above (Supplementary Fig. 11c). Parameter values are listed in Supplementary Table 3.

Supplementary Table 3. Sensory neuron parameters.

Sensory neuron parameters	Value	Description
α_{ASEL}	50 s^{-1}	ASEL depolarization rate
β_{ASEL}	8 s^{-1}	ASEL leak rate
γ_{ASEL}	1 s^{-1}	ASEL rectification (repolarization) rate
$\delta_{\mathrm{ASEL}}^{\mathrm{ON}}$	0.006 s^{-1}	ASEL desensitization rate
$\delta_{\mathrm{ASEL}}^{\mathrm{OFF}}$	0.004 s^{-1}	ASEL desensitization relaxation rate
D_{ASEL}	1	ASEL gain adaptation factor
α_{ASER}	0.25 s^{-1}	ASER activation rate
β_{ASER}	1 s^{-1}	ASER leak rate
γ_{ASER}	0.05 s^{-1}	ASER rectification rate
$\delta_{\mathrm{ASER}}^{\mathrm{ON}}, \delta_{\mathrm{ASER}}^{\mathrm{OFF}}$	0 s^{-1}	ASER desensitization rates (off)
λ	0.01 s^{-1}	ASER gain adaptation relaxation rate
α_{ASH}	0.1 s^{-1}	ASH activation rate
β_{ASH}	0.6 s^{-1}	ASH leak rate
γ_{ASH}	0.01 s^{-1}	ASH rectification rate
$\delta_{\mathrm{ASH}}^{\mathrm{ON}}, \delta_{\mathrm{ASH}}^{\mathrm{OFF}}$	0 s^{-1}	ASH desensitization rates (off)
D_{ASH}	1	ASH gain adaptation factor
C_{min}	1 mM	Unit scaling factor

In our data (supported by Oda *et al.*[14]) ASEL showed slow desensitization and ASER slow sensitization (main text, Figs. 1 and 2). Specifically, ASEL failed to respond to an increase in NaCl concentration after a pre-exposure of around 600 s. However, after such a pre-exposure ASEL did respond to larger increases in NaCl concentration (main text, Fig. 2c). This response hints at an adaptive threshold and, together with the wide dynamic range of responses, is reminiscent of Fechner's law, typically expressed as $r \propto \log s$, where r and s denote the response and signal, respectively. The incremental form of the law $\Delta r \propto \frac{\Delta s}{s}$ describes the minimal stimulus change needed to evoke a response, relative to some baseline[15]. Accordingly, in our model, the slow sensory

adaptation of ASEL to NaCl is implemented with a slowly evolving concentration sensitivity threshold, C_0 (Supplementary Fig. 10, 11)

$$\frac{\mathrm{d}C_0}{\mathrm{d}t} = \delta_i^{\mathrm{ON}} C_{\log} - \delta_i^{\mathrm{OFF}} C_0 \ . \qquad \text{(E8)}$$

In addition, the fast component F integrates over the logarithm of the NaCl concentration, capturing the wide dynamic range of behavioral responses to NaCl

$$C_{\log} = \log\left(1 + C_{(x,y,t)}/C_{\min}\right)/\log\left(1 + (C_{\max}/C_{\min})\right), \qquad \text{(E9)}$$

where $C_{\min}$ is a unit scaling factor, that imposes an effective lower cut-off to the sensory dynamic range, grossly representing the minimal concentration that can evoke a behavioral response[16]. Note that in our model, threshold adaptation only applies to ASEL, thus only there δ_i^{ON} and δ_i^{OFF} are nonzero.

In contrast to the slow desensitization of ASEL, we and Oda *et al.*[14] have shown that ASER *sensitizes* over time: longer NaCl pre-exposure results in a stronger depolarizing response to decreases in NaCl concentration (main text, Fig. 1). This sensitization is captured in our model with a multiplicative gain parameter, D_{ASER}: a slowly evolving measure of the presence of NaCl such that the fast component F increases with the pre-exposure duration, as measured experimentally (Supplementary Fig. 11). The gain parameter evolves according to

$$\frac{\mathrm{d}}{\mathrm{d}t} D_{\mathrm{ASER}} = \lambda C_{\log}(1 - D_{\mathrm{ASER}}) - \lambda D_{\mathrm{ASER}}. \qquad \text{(E10)}$$

The initial activities of the neurons are all set to rest (0) at $t = 0$. Further details of the initial conditions are given below.

ASH recruitment

Recruitment of the ASH sensory neuron is modeled as a stochastic switch between on and off states, with switching rates that vary with the animal's NaCl exposure history. In its off or *unrecruited* state, ASH is assumed to respond only to dangerous concentrations of NaCl (>300 mM), whereas in its on or *recruited* state, it responds to low concentrations of NaCl as well. Specifically, in the recruited state, the ASH sensory neuron is modeled as identical to ASER, up to a sign reversal.

Let ρ denote the propensity of ASH to be recruited, which varies monotonically from 0 (no NaCl) to 1 (saturated exposure to NaCl). The recruitment rate evolves according to

$$\frac{\mathrm{d}\rho}{\mathrm{d}t} = \kappa\left(\frac{C_{\log}}{C_{\max}} - \rho\right). \qquad \text{(E11)}$$

Hence, ρ converges to the ratio of the current concentration and a ‘maximum' concentration with a rate, κ. Let the transition rates α_{rec} and β_{rec} denote the recruitment and ‘unrecruitment’ rates respectively, such that

$$\mathrm{OFF} \underset{\beta_{\mathrm{rec}}}{\overset{\alpha_{\mathrm{rec}}}{\rightleftharpoons}} \mathrm{ON}, \qquad \text{(E12)}$$

with $\alpha_{\mathrm{rec}} = \frac{\rho}{\tau_\alpha}$ and $\beta_{\mathrm{rec}} = \frac{1-\rho}{\tau_\beta}$. The steady state probabilities $P^*(\mathrm{ON})$ and $P^*(\mathrm{OFF})$ for occupying the on (recruited) and off (unrecruited) states are then given by

$$P^*(\mathrm{ON}) = \frac{\alpha_{\mathrm{rec}}}{\alpha_{\mathrm{rec}} + \beta_{\mathrm{rec}}} \qquad \text{(E13)}$$

$$P^*(\mathrm{OFF}) = \frac{\beta_{\mathrm{rec}}}{\alpha_{\mathrm{rec}} + \beta_{\mathrm{rec}}}. \qquad \text{(E14)}$$

The above formulation of the recruitment process allows for a separation of time scales which is not needed here, so, for simplicity and parsimony, we simplify it by collapsing all three timescales to one, such that $\tau_\alpha = \tau_\beta = 1/\kappa$. Default parameter values are given in Supplementary Table 4.

We note that ASH recruitment is unlikely to be as simple as an instantaneous and stochastic on-off switch. However, modeling the recruitment kinetics in greater detail would require sufficiently resolved kinetic data (on the fraction of responders as a function of time and concentration). Our data showed a minority of responders to low NaCl concentrations without pre-exposure, and a very high fraction of responders after 600 sec exposure. Without a clear indication of a change in the response amplitude, we opted for a parsimonious binary (on-off) model of ASH responsiveness (to low NaCl concentrations) and our model of the recruitment kinetics aimed only to capture the observed statistics with minimal assumptions. Modeling the recruitment of the ASH sensory neuron as a stochastic switch between on and off states sufficed for capturing the fractions of recruited ASH neurons on salt, but alternative (e.g. continuous) models are possible. Switching rates in this model vary with the animal's NaCl exposure history (a necessary assumption). In animals that have not been pre-exposed to NaCl, ASH is in its ‘off ‘ or unrecruited state. It is implicitly assumed that in its unrecruited state, ASH neurons respond only to dangerous concentrations of NaCl. In animals that were pre-exposed to NaCl, ASH is in its ‘on’ or recruited state, hence responding to low concentrations of NaCl as well.

Supplementary Table 4. ASH recruitment parameters.

ASH recruitment	Value	Description
$C_{\max}$	100 mM	NaCl saturation level for recruitment ($\rho \to 1$)
κ	0.001 s^{-1}	Common rate parameter for recruitment kinetics

Motor system

Undulations and steering are modeled by a symmetric oscillator motif, consisting of reciprocal inhibitory and delayed excitatory connections (Supplementary Fig. 9). We used *hidden* interneurons to create a delayed connection between motor neurons. Thus, the delayed excitatory connection from VMN to DMN is implemented as two connections, one from VMN to the hidden interneuron, and another from the hidden interneuron to DMN, using the neuronal time constant of the hidden interneuron, τ_m, as a synaptic delay. The reciprocal connection from DMN to VMN is identical. The model contains two separate motor outputs: undulations and pirouettes. Both can be modulated by sensory inputs.

Undulations

Two motor neurons (VMN, DMN) in a half-center oscillator like configuration (Supplementary Fig. 9) are capable of generating and maintaining stable oscillations as well as to steer the worm (Supplementary Fig. 12).

The reciprocal connectivity pattern is reminiscent of connectivity found in several classes of head motor neurons in *C. elegans*. Compared to more compact models, e.g. Izquierdo and Lockery[2], our approach allows for the modulation of the undulation frequency as well the amplitude, more closely

matching observed trajectories of worms in the choice assay, especially in the vicinity of the quadrant boundaries (Supplementary Fig. 12).

Supplementary Fig. 12. Model worm trajectories and motor neuron activities.

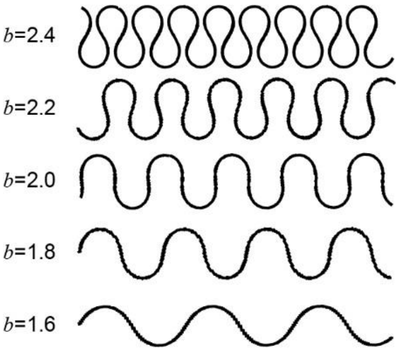

The neuronal gain parameter b determines the head undulatory trajectory, with $b = 2$, giving the realistic trajectory. We set the head speed v to obtain an overall speed of 0.1 mm/s resulting in about 23 undulations per cm at a frequency of 0.23 Hz.

In the absence of sensory input, the motor circuit will produce stable oscillations, facilitated by fast reciprocal inhibition that is released by the delayed reciprocal excitation (Supplementary Fig. 9). Any activity in one of the oscillating neurons will cause fast inhibition of the other followed by slower excitation and subsequently inhibition of the originally active neuron. Thus, the frequency and amplitude of the oscillations are determined by the timescales of the neurons, τ_{m}, the connection strengths of the reciprocal inhibition, and delay in the delayed reciprocal connections. In our model, τ_{m}, remained fixed, leaving the three pairs of reciprocal connection strengths as parameters to tune the oscillator.

Since the circuit configuration and all parameters are symmetric, symmetry breaking is required to initiate oscillations. Indeed, when VMN and DMN are equally active, the circuit does not oscillate. However, any small difference in activity (or initial conditions) is amplified by the mutual inhibition. In the model, a start 'signal' V_{start} is given for a short duration τ_{start} with opposite polarity to the two motor neurons VMN and DMN, causing them to diverge in activity. Motor output parameter values are given in Supplementary Table 5.

Supplementary Table 5. Motor output parameters.

Motor output parameters	Value	Description
v	0.22 mm s^{-1}	Locomotion speed
ω	1.5 s^{-1}	Steering strength/angular speed
V_{start}	0.001	Activity of oscillator start signal
τ_{start}	0.01 s	Duration of oscillator start signal
ω_{Ω}	0.005 s^{-1}	Base pirouette rate

Similar to other point models of *C. elegans*[2,17,18], the direction of locomotion, θ, changes as a function of the difference in activity of the dorsal and ventral motor neurons:

$$\frac{\mathrm{d}\theta}{\mathrm{d}t} = \omega(V_{\mathrm{VMN}} - V_{\mathrm{DMN}})\,, \quad \text{(E15)}$$

where ω is the steering strength. Model worms move with constant velocity v along the direction vector θ according to

$$\frac{\mathrm{d}(x,y)}{\mathrm{d}t} = v(\sin\theta, \cos\theta)\,. \qquad \text{(E16)}$$

The speed was chosen so the model worm performed realistically on the assay (see Supplementary Fig. 13a).

Pirouettes

Instant turning events are executed by resetting the orientation of the movement of the point worm. The probability of a pirouette per unit time P_Ω is encoded by the activation of the pirouette command unit V_Ω and given by a smooth and monotonically decreasing function

$$P_\Omega(V_\Omega) = w_\Omega \exp(-V_\Omega), \qquad \text{(E17)}$$

where w_Ω is the base pirouette rate. The parameters were chosen such the base rate is approximately 2.1 pirouettes per minute, and suppression of pirouettes (for sufficiently hyperpolarized values of the neuron) is possible.

When a pirouette is executed, the heading, θ, is instantaneously set to a random angle drawn from a uniform distribution.

The Model Choice Assay

Simulations are performed on a virtual two-dimensional 20 cm plate, which animals cannot leave. Upon reaching the edge of the plate, virtual animals are reoriented to a random angle drawn from a uniform distribution. Reorientation can occur multiple times in succession if worms continue to hit the edge. A choice assay (Supplementary Fig. 13) was implemented to reproduce an experimental assay designed to test naive and conditioned responses to NaCl[5]. Supplementary movies 1-14 show 500 simulated ASH-virtual ablated worms, with/without (de)sensitization in ASEL or ASER, ASEL or ASER-virtual ablated, and/or ablating the synaptic connections to the steering circuit or to the pirouette neuron.

Supplementary Fig. 13. The choice assay used in our simulations.

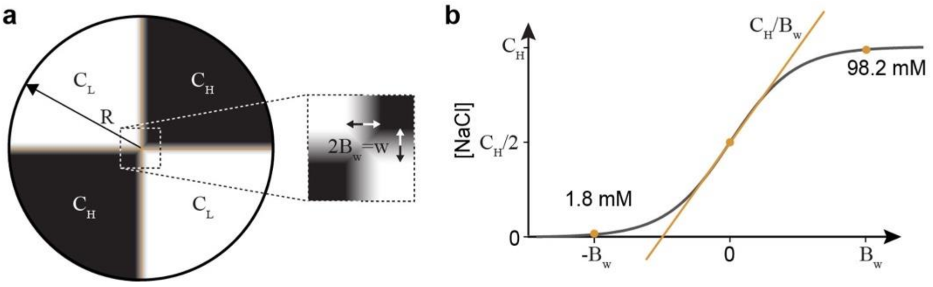

a A plate of radius R is prepared with alternating quadrants of uniform high (C_H=100 mM, black), and low (C_L=0 mM, white) NaCl concentrations. **b** Between quadrants, a sigmoidal concentration profile is imposed such that 96% of the concentration range is spanned within 4 mm of the interface.

We model the virtual quadrant assay on a Cartesian coordinate system whose origin is at the center of the arena, at the intersection between the four quadrants. The concentration field $C(x, y)$ ranges between zero and a maximum, C_{H}. It is modeled using a hyperbolic tangent function

$$C(x, y) = \frac{C_{\mathrm{H}}}{2}\left[1 + \mathrm{sgn}(x \cdot y)\tanh\left(\frac{d_{\mathrm{ax}}(x,y)}{5B_{\mathrm{w}}}\right)\right], \quad \text{(E18)}$$

which results in a realistic transition and close to zero salt gradients within the quadrants (Supplementary Fig. 13). Here $d_{\mathrm{ax}}(x, y)$ denotes the distance from the nearest quadrant interface, defined as $\min(|x|, |y|)$ with interfaces at $x = 0$ and at $y = 0$ and sgn is the sign function.

Choice assay experiments were performed with both naive animals and conditioned animals. In these experiments, animals were washed in either salt-free or salt-containing CTX buffer for 15 minutes before being placed on the assay plate. To mimic pre-exposure in our model, we simulated animals on a plate with a uniform concentration of salt for 15 minutes, and then virtually transferred them to the center of the quadrant plate; when the concentration decrease is encountered, we manually switched on (i.e., recruited) ASH.

As in the experimental protocol, the result was quantified using a chemotaxis index (CI) by counting the number of worms after 600 seconds in each quadrant. In the experiments, worms that remained trapped near a border between quadrants were excluded, and not counted towards the CI. As this does not apply to the model, we did not exclude any region of the plate. To ensure that this does not alter our results, we verified that excluding a region around the border produces statistically identical CIs. Parameter values of the choice assay are given in Supplementary Table 6.

Supplementary Table 6. Virtual choice-assay variable initialization and simulation parameters (naïve animals).

Variable/Parameter	Value	Description
N	1,000	Number of worms simulated
τ_{end}	600 s	Duration of the assay
$\mathrm{d}t$	0.1 ms	Integration time step of the simulation
$\theta(0)$	Random	Initial direction drawn from uniform distribution
C_{H}	100 mM	NaCl concentration in NaCl quadrants
B_{w}	0.2 cm	Half width of boundary region with NaCl gradient
V_i	0	Initial activity for all neurons
F_i	0	Initial value for fast component of all sensory neurons
S_i	0	Initial value for slow component of all sensory neurons
C_0	0 mM	Initial value for the baseline of ASEL
D_{ASER}	0	Initial value for fast component in ASER
C_{wash}	100 mM	Concentration of NaCl in buffer used for conditioning

Population models and simulations

Population models were used to test the robustness of our models and the effect of adaptation on robustness in the quadrant assay. Each population consisted of model animals either with (test) or without (control) ASE adaptation. To generate population models of animals, we perturbed the

parameters, α_{ASEL}, α_{ASER}, β_{ASEL}, β_{ASER} and γ_{ASEL}, γ_{ASER}, associated with ASEL and ASER responses (6 parameters in total), by independently subjecting each parameter in each animal to noise. Specifically, model parameters were perturbed by applying multiplicative noise with noise levels independently sampled from a lognormal distribution. In other words, we substituted each parameter p_{i} by $p_{\mathrm{i}}\ \mathrm{e}^{\sigma x}$ where x was sampled from a normal distribution with standard deviation σ (see Supplementary Fig. 7a). To focus on the role of adaptation, in these simulations ASH was inactive. We simulated each model animal across each population (with $n = 500$) and compared the CI achieved after 10 minutes of simulation of the quadrant assay for different populations of animals. Noise amplitudes varied from low (σ =0.01) to very high noise levels (σ =1.28), in which the CI performance falls sharply in both control and test animals.

The Model Spot Assay

Simulations were performed in a virtually infinite assay with NaCl spots of standard normal (Gaussian) concentration profiles (Supplementary Fig. 14). We quantified the exploration behavior in terms of hops from one spot to another. For this purpose, the region within standard deviation to a center of a spot is considered “inside” the spot (Supplementary Fig. 14). For each trajectory in the spot assay, we have collected the times the trajectory enters the inside of a spot for the first time after leaving another spot. Supplementary movies 15-30 show 100 (out of 500) simulated worms, wild-type, ASH-virtual ablated, or no (de) sensitization in ASEL or ASER, on the spot assay, with a peak concentration of 100 or 200 mM NaCl and spot separation distance of 3.3 or 5 cm.

Supplementary Fig. 14. The model spot assay.

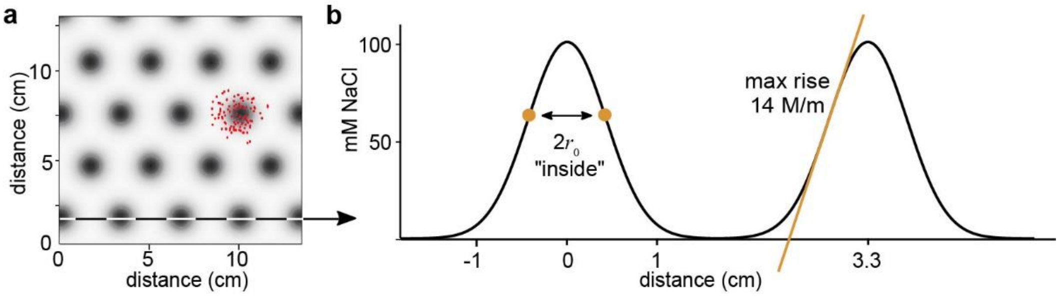

a A plate of virtually infinite size is prepared with NaCl spots (in black) arranged in a hexagonal grid. Animals are indicated as red dots. **b** The NaCl concentration at a point $p = (x, y)$ is the sum of contributions $\sum_{\mathrm{spots},i} s\,(|p - q_i|)$ from salt spots, with each salt spot following a Gaussian radial concentration profile around a center, denoted $q_i = (x_i, y_i)$, $s(r) = C_{\mathrm{S}} \exp(-2(r/r_0)^2)$ (assuming radial diffusion is well approximated by linear diffusion from a point). An infinite virtual assay is constructed by making use of the periodicity of the spot positions. At any point, p, we approximate the salt concentration by a superposition of the contributions from the seven nearest spots (verified to yield values within machine precision of the exact concentration value).

Supplementary Table 7. Virtual spot assay parameters.

Initialization (naïve)	Value	Description
τ_{end}	3600 s	Duration of the assay
$\mathrm{d}t$	0.1 ms	Integration time step of the simulation
$\theta(0)$	Random	Initial direction (sampled from a uniform distribution)
C_{S}	100 mM	Peak NaCl concentration (at each spot center)
r_0	0.6 cm	Spot radius ([NaCl] standard deviation)

Supplementary References

1 Ghosh D. D., Sanders T., Hong S., McCurdy L. Y., Chase D. L., Cohen N., Koelle M. R., Nitabach M. N. (2016). Neural Architecture of Hunger-Dependent Multisensory Decision Making in *C. elegans*. *Neuron* **92**, 1049-1062. doi: 10.1016/j.neuron.2016.10.030

2 Izquierdo, E. J., Lockery, S. R. (2010). Evolution and analysis of minimal neural circuits for klinotaxis in *Caenorhabditis elegans*. *J. Neurosci.* **30**, 12908-12917. doi: 10.1523/JNEUROSCI.2606-10.2010

3 Suzuki, H., Thiele T. R., Faumont S., Ezcurra M., Lockery S. R., Schafer W. R. (2008). Functional asymmetry in *Caenorhabditis elegans* taste neurons and its computational role in chemotaxis. *Nature* **454**, 114-117. doi: 10.1038/nature06927

4 Thiele, T. R., Faumont, S., Lockery, S. R. (2009). The neural network for chemotaxis to tastants in *Caenorhabditis elegans* is specialized for temporal differentiation, *J. Neurosci.* **29**, 11904-11911. doi: 10.1523/JNEUROSCI.0594-09.2009

5 Jansen, G., Weinkove, D., Plasterk, R. H. (2002). The G-protein gamma subunit *gpc-1* of the nematode *C. elegans* is involved in taste adaptation, *EMBO J.* **21**, 986-994. doi: 10.1093/emboj/21.5.986

6 Hukema, R. K., Rademakers, S., Dekkers, M. P., Burghoorn, J., Jansen, G. (2006). Antagonistic sensory cues generate gustatory plasticity in *Caenorhabditis elegans*. *EMBO J* **25**, 312-322. 10.1038/sj.emboj.7600940

7 Hukema, R. K., Rademakers, S. and Jansen, G. (2008). Gustatory plasticity in *C. elegans* involves integration of negative cues and NaCl taste mediated by serotonin, dopamine, and glutamate. *Learn. Mem.* **15**, 829-836. doi: 10.1101/lm.994408

8 Iino, Y., Yoshida, K. (2009). Parallel use of two behavioral mechanisms for chemotaxis in *Caenorhabditis elegans*. *J. Neurosci.* **29**, 5370-5380. doi: 10.1523/JNEUROSCI.3633-08.2009

9 Larsch, J., Ventimiglia, D., Bargmann, C. I., Albrecht, D. R. (2013). High-throughput imaging of neuronal activity in *Caenorhabditis elegans*. Proc. Natl. Acad. Sci. USA, 110: E4266-E4273; doi:10.1073/pnas.1318325110

10 White, J. G. Southgate, E. Thomson, J. N., Brenner, S. (1986). The structure of the nervous system of the nematode *C. elegans. Philos. Trans. R. Soc. Lond. B Biol. Sci.***314**:1–340. doi: 10.1098/rstb.1986.0056

11 Liu, Q., Hollopeter, G., Jorgensen, E. M. (2009). Graded synaptic transmission at the *Caenorhabditis elegans* neuromuscular junction, *Proc. Natl. Acad. Sci. USA* **26**:10823-10828. doi: 10.1073/pnas.0903570106

12 Hilliard, M. A., Apicella A. J., Kerr R., Suzuki H., Bazzicalupo P., Schafer W. R. (2005). *In vivo* imaging of *C. elegans* ASH neurons: cellular response and adaptation to chemical repellents. *EMBO J.* **24**, 63-72. doi: 10.1038/sj.emboj.7600493

13 Kato, S., Xu, Y., Cho, C. E., Abbott, L. F., Bargmann, C. I. (2014). Temporal responses of *C. elegans* chemosensory neurons are preserved in behavioral dynamics. *Neuron* **81**, 616-628. doi: 10.1016/j.neuron.2013.11.020

14 Oda, S., Tomioka, M., Iino, Y. (2011). Neuronal plasticity regulated by the insulin-like signaling pathway underlies salt chemotaxis learning in *Caenorhabditis elegans*. *J. Neurophysiol.* **106**, 301-308. doi: 10.1152/jn.01029.2010

15 Cohen, N., Sanders, T. (2014). Nematode locomotion: dissecting the neuronal-environmental loop. *Curr. Opin. Neurobiol.* **25**, 99-106. doi: 10.1016/j.conb.2013.12.003.

16 Ward, S. (1973). Chemotaxis by the nematode *Caenorhabditis elegans*: identification of attractants and analysis of the response *Proc. Natl. Acad. Sci. U.S.A.* **70**:817–821. doi: 10.1073/pnas.70.3.817

17 Bryden, J.A, Cohen, N. (2008). Neural control of *Caenorhabditis elegans* forward locomotion: the role of sensory feedback. *Biol. Cybern.* **98**, 339-351. doi: 10.1007/s00422-008-0212-6

18 Bryden, J.A, Cohen, N. (2004). A simulation model of the locomotion controllers for the nematode *Caenorhabditis elegans*. *From Animals to Animats* **8**, 183-192. ISBN 978-0-262-69341-7
