# Supplementary material for: Plasticity in gustatory and nociceptive neurons controls decision making in C. elegans salt navigation
Source: Commun Biol. 2021 Sep 9;4:1053. doi: 10.1038/s42003-021-02561-9 (PMC8429449; doi:10.1038/s42003-021-02561-9)
Supplement: Supplementary file 3 — Description of Additional Supplementary Files [file 42003_2021_2561_MOESM3_ESM.pdf]

Description of Additional Supplementary Files

File name: Supplementary Movies

Description:

Supplementary Movies **1-14** show 500 simulated worms for 10 minutes on the quadrant choice assay, with upper right and lower left quadrants containing 100 mM NaCl, and the other two quadrants contain no NaCl. Corresponds to Figure 7.

Supplementary Movie 1: ASH ablated
Supplementary Movie 2: ASH ablated, no (de)sensitization in ASEL or ASER
Supplementary Movie 3: ASH ablated, no connection between ASEL and steering
Supplementary Movie 4: ASH ablated, no connection between ASEL and pirouettes
Supplementary Movie 5: ASH ablated, no connection between ASER and steering
Supplementary Movie 6: ASH ablated, no connection between ASER and pirouettes
Supplementary Movie 7: ASH ablated, ASEL ablated
Supplementary Movie 8: ASH ablated, ASER ablated
Supplementary Movie 9: ASH ablated, no steering
Supplementary Movie 10: ASH ablated, ASEL ablated, no steering
Supplementary Movie 11: ASH ablated, ASER ablated, no steering
Supplementary Movie 12: ASH ablated, no pirouettes
Supplementary Movie 13: ASH ablated, ASEL ablated, no pirouettes
Supplementary Movie 14: ASH ablated, ASER ablated, no pirouettes

Supplementary Movies **15-18** show 100 (out of 500) simulated worms on the spot assay, with a peak concentration of 100 mM NaCl and spot separation distance of 3.3 cm. Corresponds to Figure 9.

Supplementary Movie 15: wild type
Supplementary Movie 16: ASH ablated
Supplementary Movie 17: no (de)sensitization in ASEL or ASER
Supplementary Movie 18: ASH ablated, with no (de)sensitization in ASEL or ASER

Supplementary Movies **19-22** show 100 simulated worms on the spot assay, with a peak concentration of 200 mM and spot separation distance of 3.3 cm. Corresponds to Figure 9.

Supplementary Movie 19: wild type
Supplementary Movie 20: ASH ablated
Supplementary Movie 21: no (de)sensitization in ASEL or ASER
Supplementary Movie 22: ASH ablated, with no (de)sensitization in ASEL or ASER

Supplementary Movies **23-26** show 100 simulated worms on the spot assay, with a peak concentration of 100 mM and spot separation distance of 5.0 cm. Corresponds to Figure 9.

Supplementary Movie 23: wild type
Supplementary Movie 24: ASH ablated
Supplementary Movie 25: no (de)sensitization in ASEL or ASER
Supplementary Movie 26: ASH ablated, with no (de)sensitization in ASEL or ASER

Supplementary Movies **27-30** show 100 simulated worms on the spot assay, with a peak concentration of 200 mM and spot separation distance of 5.0 cm. Corresponds to Figure 9.

Supplementary Movie 27: wild type
Supplementary Movie 28: ASH ablated
Supplementary Movie 29: no (de)sensitization in ASEL or ASER
Supplementary Movie 30: ASH ablated, with no (de)sensitization in ASEL or ASER

File name: Supplementary Data 1
Description: Source data underlying figure 1

File name: Supplementary Data 2
Description: Source data underlying figure 2

File name: Supplementary Data 3
Description: Source data underlying figure 3

File name: Supplementary Data 4
Description: Source data underlying figure 4

File name: Supplementary Data 5
Description: Source data underlying figure 5

File name: Supplementary Data 6
Description: Source data underlying figure 6

File name: Supplementary Data 7
Description: Source data underlying figure 7

File name: Supplementary Data 8
Description: Source data underlying figure 8

File name: Supplementary Data 9
Description: Source data underlying figure 9
